# Cooled Radiofrequency Ablation of Thoracic Medial Branches for the Treatment of Chronic Thoracic Pain

**DOI:** 10.3390/healthcare13121468

**Published:** 2025-06-18

**Authors:** Alaa Abd-Elsayed, Alessandro Preda, Barnabas T. Shiferaw, Alexis K. Harrell, Kenneth J. Fiala

**Affiliations:** 1 Department of Anesthesiology, University of Wisconsin School of Medicine and Public Health, 600 Highland Avenue, B6/319 CSC, Madison, WI 53792, USA; apreda@wisc.edu (A.P.); bshiferaw@wisc.edu (B.T.S.); kenfiala1@gmail.com (K.J.F.); 2 Department of Natural Sciences and Mathematics, Spelman College, Atlanta, GA 30314, USA; alexis.harrell3755@gmail.com

**Keywords:** radiofrequency ablation, thoracic pain, chronic pain

## Abstract

**Background:** Chronic thoracic pain is commonly caused by thoracic facet dysfunction, intercostal neuralgia, surgery, or thoracic pain secondary to cancer and is present in approximately 15% of the population. Conventional treatments, including pharmacotherapy and transcutaneous electrical nerve stimulation, are often ineffective and are often associated with poorly tolerated adverse effects. Cooled radiofrequency ablation (c-RFA) is a minimally invasive procedure that uses radiofrequency energy delivered through a probe to lesion the targeted nerve and provide significant and lasting relief. **Methods**: This study was a retrospective analysis of data extracted from UW-Health Electronic Medical Health records from October 2015 through June 2024. Patient data were collected, including diagnosis, pre-operative pain score, post-operative pain score, duration of relief, age, sex, and BMI. A two-tailed paired *t*-test was used to analyze the pre-operative and post-operative pain scores. A *p*-value < 0.05 was considered significant. **Results**: A total of 111 thoracic c-RFA procedures were reviewed; 43 were excluded due to absent pre-operative or post-operative pain scores in medical records. A total of 68 procedures were included in the analysis, comprising 55 patients: 25 females and 30 males with an average age of 51.31 ± 18.22 years and a BMI of 29.79 ± 6.48 kg/m^2^. Improvement in pain scores was reported in 77.94% (n = 53), 16.18% (n = 11) reported no change, and 5.88% (n = 4) reported worsening pain. Patients reported an average pre-operative pain score of 5.98 (M = 5.98, SD = 1.91) and an average post-operative pain score of 3.06 (M = 3.06, SD = 2.52); this achieved significance (*p* < 0.0001). Of the 77.94% (n = 53) charts that noted improvement, there is an average of 62.83 ± 28.48% reduction from their pre-operative pain scores. The average duration of relief lasted 11.85 ± 13.42 months. **Conclusions**: This study supports the efficacy and safety of c-RFA as a minimally invasive therapy for chronic thoracic pain refractory to conservative measures.

## 1. Introduction

Chronic thoracic pain is defined as persistent upper and middle back pain that lasts six months or beyond and is most commonly caused by thoracic facet dysfunction, intercostal neuralgia, surgery, or thoracic pain secondary to cancer and is present in approximately 15% of the population [1,2,3,4,5,6].

Conventional treatment for thoracic pain includes NSAIDS, acetaminophen, physical therapy, antidepressants, anticonvulsants, transcutaneous electrical nerve stimulation, thoracic nerve blockades, and opioids [7,8,9,10,11,12]. These treatments often provide limited pain relief and can be associated with poorly tolerated side effects profiles including weight gain and decreased libido associated with antidepressants; sedation and dry mouth associated with anticonvulsants; and addiction, abuse potential, and overdose risk associated with opioids [8,13,14].

Cooled radiofrequency ablation (c-RFA) is a promising, minimally invasive therapy that has been effective in treating chronic pain and pain in other parts of the body [15,16,17]. Radiofrequency ablation, a form of thermal ablation first described in 1960 and introduced in the 1970s, has evolved significantly since its inception. It is now a viable, minimally invasive procedure that can be employed as an effective treatment for chronic pain in other parts of the body such as the cervical spine and knee [15,16,18,19,20].

Conventional RFA utilizes a high-frequency alternating current to produce thermal energy, which is delivered through a specialized probe to create precise neurolytic lesions on nociceptive fibers responsible for transmitting pain from the affected region [21,22]. Conventional RFA probe tips are set at 80 °C to 90 °C for 90 s [18]. In this study, cooled radiofrequency ablation, a specific type of RFA, was used. c-RFA employs a similar technique to traditional RFA but allows for a larger treatment zone, preventing blood coagulation and tissue charring by intermittently cooling the electrode tip. c-RFA is assisted by imaging such as fluoroscopy or ultrasound to allow for visualization of the needle into the targeted area before starting the ablation [5]. In c-RFA, the probe is heated to 60 °C and the targeted tissue reaches temperatures up to 80 °C, creating a lesion on the targeted nerve, resulting in interrupted transmission of pain signals to the brain [15].

This retrospective analysis investigates the efficacy of c-RFA as a treatment for chronic thoracic pain refractory to conservative measures.

## 2. Methods

This study is a retrospective analysis of data collected from patients having previously undergone cooled radiofrequency ablation (c-RFA) of thoracic nerves as a treatment for chronic thoracic pain between 2015 and 2024 at the University of Wisconsin Hospital and Clinics. Comprehensive data collection included the patients’ demographics such as age, sex, body mass index (BMI), and relevant past medical history. Patient diagnosis was determined by reviewing patients’ past medical history and indications for c-RFA. Patients’ diagnoses included thoracic facet arthropathy, lumbar facet arthropathy, cervical facet arthropathy, thoracic spondylosis, lumbar spondylosis, costovertebral joint pain, thoracic pain, scoliosis, post-laminectomy syndrome, and paraspinal posterior chest wall pain. The patient’s diagnosis, pre- and post-operative pain scores, subjective percent pain relief, duration of improvement, along with any adverse events were reported.

### 2.1. Patient Selection and Diagnostic Block

All patients included in this study had chronic thoracic pain syndromes refractory to conservative treatment. Patients were excluded if they were not able to provide follow-up pain scores after the procedure or received an additional procedure at the RFA site such as steroid injections. Before proceeding with radiofrequency ablation, patients were required to undergo two diagnostic blocks of the thoracic nerves. After obtaining written informed consent, patients were taken to the procedure room. Pre-procedure blood pressure and pulse were stable and recorded in the patients’ chart. The patients were placed in a prone position and were prepped with 2% chlorhexidine gluconate and 70% isopropyl alcohol and draped in a sterile fashion. The skin was infiltrated with 1% lidocaine at the junction of the transverse process with the vertebral body at the respective thoracic level. A 22-gauge, 3.5-inch needle was inserted into the thoracic medial branch under radiographic guidance. Following placement of the needle and negative aspiration, 1 cc of bupivacaine 0.25% was injected at each level. After the procedure, the needles were removed. The skin was cleaned and a sterile dressing was applied. Following the procedure, the patients’ vital signs were assessed and they were discharged home in good condition after being given discharge instructions. A greater than 50% reduction in pain score from the diagnostic nerve blocks was required prior to moving forward to radiofrequency ablation.

### 2.2. Cooled RFA Procedure

After obtaining written informed consent, the patient was taken to the procedure room. Pre-procedure blood pressure and pulse were stable and recorded in the patient’s clinic chart. The patients were placed in a prone position and the desired target locations were prepped with 2% chlorhexidine gluconate and 70% isopropyl alcohol and draped in a sterile fashion. A formal time-out was performed to identify the correct patient and to review the correct procedure, anticoagulation status, allergies, and correct sites and sides. The skin over the respective thoracic medial branches was identified by fluoroscopy. An amount of 1% lidocaine was used to anesthetize the skin. Then, a 17-gauge, 50 mm stimulating needle with a 4 mm active tip was placed under fluoroscopic guidance at the site of the medial branches at respective thoracic levels. Lateral imaging confirmed the appropriate depth on the articular column and negative aspiration was confirmed. Motor testing was then performed at 2 Hz. An amount of 1.5 mL of 2% preservative-free lidocaine was then injected at each level after negative aspiration. The c-RFA was then carried out in lesion mode. The settings were 80 °C tissue temperature, lesion mode, and 150 s. The skin was then cleaned, and a sterile dressing was applied for each patient.

### 2.3. Follow-Up

Following their procedure, patients returned for follow-up appointments to discuss their post-operative status, any adverse effects, and report their post-operative pain scores.

### 2.4. Pain Scoring

Pre-operative and post-operative pain scores were obtained using the visual analog scale (VAS) and ranged from 0/10 to 10/10. The VAS was used due to its reliability and validity as a measure of chronic pain in clinical research [23]. Pre-operative pain scores were obtained from the pre-operative visit or immediately prior to the diagnostic block. Post-operative pain scores were recorded from the patient’s pain diary or directly from the patients at their scheduled post-operative visit if the pain diary was not available. If a post-operative pain VAS score was not obtained but if a patient reported improvements such as 20% or 50% improvement in their pain, then a post-operative pain score was calculated. For example, if a patient had a pre-operative pain score of 8/10 and reported a 50% improvement in their pain, a post-operative pain score was calculated to be 4/10. If there was a discrepancy between the post-operative pain score and the percent improvement in pain reported by the patient, the post-operative percentage improvement was preferred. If patients reported a range for the pain scores, the average between the highest and lowest pain scores was calculated and reported as their pain score. Post-operative pain scores were not included if a procedure such as a steroid injection or surgery at the site of the procedure took place between the c-RFA procedure and the reporting of the post-operative pain scores.

If only a VAS score was reported then percent improvement was calculated by taking the difference between the pre-operative pain score and post-operative score, dividing by the pre-operative pain score, and multiplying by 100. Patients who reported complete resolution of pain were listed as 100% improvement. Percent improvement was calculated for patients who reported improvement in their pain or patients who received no benefit from the procedure, which was recorded as a 0% improvement.

Duration of relief was analyzed only for patients who reported an improvement in their pain or had a percent improvement greater than 0%. Adverse events were reported regardless of the finding of pre-operative or post-operative pain scores.

### 2.5. Statistical Analysis

A two-tailed paired *t*-test was used to evaluate for statistical significance between pre-RFA vs. post-RFA pain scores. A *p*-value of statistical significance was set at *p* < 0.05.

### 2.6. Ethical Approval

Institutional Review Board (IRB) exemption was obtained and compliance with patient confidentiality was kept throughout the study.

## 3. Results

In this retrospective analysis, 111 patient charts were reviewed and 43 were excluded from the analysis. A total of 42 were excluded due to failure to report either a pre-operative or post-operative pain score and 1 was excluded due to mid-procedural pain causing the procedure to be aborted. The patients included in this study consisted of 55 patients with a total of 68 procedures composed of 30 males and 25 females. The average age of patients in this study was 51.31 ± 18.22 with an average BMI of 29.79 ± 6.48 kg/m^2^ (Table 1).

The average pre-operative pain score was 5.98 ± 1.91 with an average post-operative pain score of 3.06 ± 2.52. A two-tailed paired *t*-test yielded a statistically significant difference between pre-operative and post-operative pain scores with a *p*-value < 0.0001. In total, 74.55% of patients reported an improvement in their pain scores and of these patients, they reported a 62.83 ± 28.47% improvement in their pain with an average length of improvement of 11.85 ± 13.42 months (Table 2).

Of the 111 procedures reviewed, 18 reported adverse events. Adverse events included worsening pain at the affected site, neuritis, headaches, blood pressure increase, back spasms, back tightness, nausea, and bradycardia. The most commonly reported adverse event was worsening pain, with affected patients generally experiencing an increase in their pre-operative thoracic pain following the procedure. The increased pain generally lasted about 1–2 weeks and then regressed to pre-operative levels or below. The most serious adverse event was nausea leading to the patient visiting urgent care. All adverse events resolved within 4 months of the procedure, spontaneously or with treatment (Table 3).

## 4. Discussion

This retrospective analysis provides evidence for the efficacy of c-RFA as a treatment for chronic pain secondary to thoracic arthropathy. The reduction in VAS pain scores by 62.83 ± 28.48% supports the efficacy of c-RFA for treating thoracic pain. Additionally, 74.55% of patients saw an improvement in their pre-operative and post-operative VAS scores, further indicating the utility of c-RFA in improving patient pain. We also identified a duration of relief of 11.85 ± 13.42 for effective c-RFAs. This high standard deviation is notable because of how it reflects on the efficacy of c-RFAs with 8 procedures out of the 53 (15.14%) experiencing a duration of relief of 24 months or greater with 3 procedures (5.66%) experiencing greater than 48 months of relief. A sample size of (n = 68) increases the validity and generalizability of this study, adding to the existing literature on c-RFA procedures.

The current literature for c-RFA for chronic thoracic pain is limited but a study conducted in 2020 found that 100% of patients reported pain reduction after six months (n = 40). Patients experienced a reduction of 37.58% in their numeric rating scale (NRS) scores within 6–12 months after c-RFA. Patients experienced a minimum of 30 weeks of pain relief with a maximum of 112 weeks. All patients required a repeat c-RFA procedure within a 112-week window [24]. The recurrence of pain following RFA can be for several reasons. One etiology that has been described in the literature is the anatomic variability in nerve location and variable rates of joint degeneration, which can be associated with progressive pain, increasing the need for repeat RFAs [25]. Comparatively, we found that patients experienced a 52.89% reduction in pain scores compared to a 37.58% decrease for ethanol ablation, further indicating the efficacy of the c-RFA procedure [26]. This difference may in part be due to our use of the VAS pain scale compared to their use of the NRS scale. Additionally, previous studies have shown that patients who presented higher pre-procedural pain scores were more likely to react positively to the RFA, which may contribute to the variability in patient responses [27]. The mode of lesion in this study differed slightly from our general procedure with active needle tips being 5.5 mm compared to our use of 4 mm active tips. Only one procedure experienced an adverse event, which was burning at the site of injection. Additionally, current evidence indicates that conventional radiofrequency ablation and cooled radiofrequency ablation are equally efficacious for lumbar facet-mediated pain [25]. Currently, there is limited literature on cooled radiofrequency ablation of thoracic facet-medicated pain. This article is a critical data set in investigating the role of c-RFA in treating chronic thoracic pain.

Alcohol ablation was found to have a longer duration of relief than thermal ablation when used in patients with recurrent thoracolumbar facet joint pain who had previously undergone radiofrequency ablation with a pain-free recurrence rate of 24 months and 10.7 months, respectively [25]. However, the comparability of our study and this study is limited by the selected patient base, diverse injection areas, and study methodology. Additionally, we found that there was a high recurrent efficacy of the c-RFA procedures with 100% of our patients who received recurrent treatments experiencing consistent improvement duration. Alcohol ablation treatment did not result in any significant adverse events in that study, whereas two patients in our study experienced severe adverse events resulting in visits to the emergency department. This could be due to the different inclusion and exclusion criteria, with the alcohol ablation study only including patients who had a previously successful RFA procedure while we included both patients new to c-RFA and those who had previously benefited from c-RFA.

### 4.1. Adverse Events

Adverse events were reported in 6.17% of our 111 procedures. The most commonly reported adverse event was a worsening pain in the affected area defined as an increase in VAS scores in the days following the c-RFA procedure. In most cases, the pain resolved spontaneously in the weeks following. There were five instances of neuritis that either resolved spontaneously or with a short course of steroids. One patient developed a hemangioma at the T8 vertebral body 2 days post-operatively alongside numbness in both hands with unilateral radiation to the shoulder on the right side. One month later, the patient had one episode of bilateral finger tingling, which resolved with steroids. There were two instances of headaches with one resolving spontaneously within a month. The other instance resulted in a severe adverse event requiring two visits to the emergency department. The patient was successfully treated with IV metoclopramide and oral acetaminophen resolving the issue within the week.

One patient received a c-RFA at the same level separated by two years in which both injections exacerbated the patient’s pre-existing hypertension, leading to headaches. This increase in blood pressure occurred within the week after each c-RFA. Additionally, the increase in blood pressure with headaches was noted in the second diagnostic branch block as well. This adverse effect was successfully treated by adjusting the patient’s dose of lisinopril by 10 mg per day, which was later readjusted to pre-operative levels. We hypothesize that this could be manifestations of autonomic dysreflexia, which generally occurs in spinal cord trauma above the T6 level but has been reported to occur in non-traumatic cases such as radiation myelopathy and cisplatin-induced polyneuropathy [28]. This patient has a significant medical history of paraplegia at T10 with past surgical spinal fusions of the vertebrae between the T3 and L3 levels. Combined with their reported headaches and increase in blood pressure to above 170 systolic, these are all indications of autonomic dysreflexia. There were two instances of back spasms, which either resolved spontaneously or with a Botox injection. There was one report of back tightness, which resolved with one month of physical therapy. There was one instance of nausea and lightheadedness three days following the procedure. The patient presented to urgent care and was successfully treated with tramadol, within the week. There was an instance of bradycardia during the procedure, which resolved spontaneously and had no notable lasting impact on the patient’s health.

### 4.2. Limitations

Patient follow-up was a major limitation in this study. Out of the 111 reviewed charts, 42 charts (37.84%) were excluded due to a lack of pre-operative or post-operative pain scores. Due to the retrospective nature of this analysis, strict post-operative follow-up protocols could not be implemented in this study. As patients began to experience pain relief, it is likely that patients did not feel the need to follow up in the clinic, which likely contributed to the difficulty in assessing post-operative pain scores. Due to the difficulty in scheduling a follow-up visit, the cost, and time requirements, patients could be turned off by the barriers to contributing to a post-operative VAS score. Furthermore, patients who attended a follow-up visit were not all asked about post-operative VAS scores. Whereas other patients may have been asked about their pain at these visits, many patients gave ambiguous answers that were unable to be quantified. Additionally, our observed duration of relief was skewed downwards due to the date of some procedures. With 12 procedures (22.64%) having been performed after 1 June 2023, our duration of relief for these procedures would not have been able to achieve the 11.85 month mean of duration of relief, thereby skewing the data downwards. We sought to include these data despite their negative effect for duration of relief as they allow us to accurately capture the other aspects of c-RFA such as pain scores and adverse effects. This is in contrast with the previously mentioned higher relief scores, which were obtained from completed data sets and therefore are accurate representations of pain scores, adverse effects, and duration of relief. Overall, this range indicates that further research is warranted to provide a complete picture for duration of relief.

The subjectivity of pain was also a limiting factor in the assessment of the efficacy of this study. As an inherently internal measure, quantifying across time and patients comes with validation issues. We combated this subjectivity through the use of the VAS pain rating system, which is a highly validated method of quantifying subjective pain ratings [29].

This study looked at a wide range of patients, which varied in age, BMI, and sex, and psychological comorbidities, which have the ability to confound pain scores. However, as a retrospective analysis, it is not possible to standardize these metrics, leading to limitations in the strength of our results; however, we compensated for this through our large sample size and long follow-up period.

Finally, some patients were undergoing concurrent methods of treatment, leading to difficulty in untangling the independent effects of c-RFA procedures on VAS pain scores. However, we aimed to mitigate these pitfalls by adopting a transparent process and employing a systematic approach to data collection. This was aided by the fact that the majority of our patients receive healthcare only within our health system, so data are accurately reported and organized within their charts.

## 5. Conclusions

This study supports the efficacy of c-RFA as a treatment modality for chronic thoracic pain. With a statistically significant reduction between pre-operative and post-operative VAS scores, we are able to assert that this study supports the existing literature and further validates c-RFA for the treatment of chronic thoracic pain.

## Figures and Tables

**Table 1:** Patient demographics.

Total # of procedures	68
Total # of patients	55
Sex	F = 25/M = 30
Age (years)	51.31 ± 18.22
BMI (kg/m^2^)	29.79 ± 6.48

**Table 2:** Summary of results.

VAS pain score pre-procedure	5.98 ± 1.91
VAS pain score post-procedure	3.06 ± 2.52
VAS % improvement	49.78 ± 35.81
VAS % improvement in effective RFAs	62.83 ± 28.48
Duration of improvement (Months)	11.85 ± 13.42

**Table 3:** Adverse events.

Adverse Event	Number
Worsening pain at the affected site	8
Neuritis	5
Headache	2
Back spasms	3
Nausea	1
Bradycardia	1

## Data Availability

The data generated and analyzed in this study are available from the corresponding author upon reasonable request.

## Abbreviations

The following abbreviations are used in this manuscript:

c-RFA	Cooled radiofrequency ablation
BMI	Body mass index
VAS	Visual analog scale
IRB	Institutional Review Board
NRS	Numeric rating scale
